# Effect of endotracheal tube lubrication on cuff pressure increase during nitrous oxide exposure: a laboratory and prospective randomized controlled trial

**DOI:** 10.1186/s12871-019-0837-0

**Published:** 2019-08-31

**Authors:** Moriyoshi Oji, Yukihide Koyama, Hiroyuki Oshika, Masashi Kohno, Yusuke Nakahashi, Sayano Fukushima, Hidemasa Iwakura, Tomio Andoh

**Affiliations:** 1 Department of Anesthesia, Tomei Atsugi Hospital, Atsugi, Japan; 2 Department of Anesthesiology, St. Marianna University School of Medicine, Kawasaki, Japan; 3 Department of Anesthesiology, Mizonokuchi Hospital, Teikyo University School of Medicine, 5-1-1 Futako, Takatsu-ku, Kawasaki, Kanagawa Prefecture 213-8507 Japan

**Keywords:** K-Y™ jelly, Lubrication, Endotracheal tube cuff, Cuff pressure increase, Nitrous oxide diffusion

## Abstract

**Background:**

We previously demonstrated that lubrication of an endotracheal tube (ETT) cuff with K-Y™ jelly strongly and significantly inhibited the increase in cuff pressure during nitrous oxide (N_2_O) exposure in vitro. However, in our previous study, we identified critical differences between some influential factors, such as the amount of lubricant retained on the cuff, and studied temperature differences between laboratory and clinical conditions. Therefore, it remained unclear whether this effect holds true in clinical settings.

**Methods:**

We first sought to study how changes in the amount of K-Y™ jelly and temperature influence the inhibitory effects of the lubricant on the increase in N_2_O-induced cuff pressure in vitro. Furthermore, we aimed to determine whether the application of K-Y™ jelly inhibits the increase in ETT cuff pressure during general anesthesia using N_2_O in adult patients.

**Results:**

In the laboratory studies, we found that K-Y™ jelly inhibited the cuff pressure increase dose-dependently when the dose of K-Y™ jelly was varied (*P* = 0.02), and that such an inhibitory effect decreased with an increase in the studied temperature (*P* = 0.019). In the clinical study, lubrication with K-Y™ jelly slightly, but significantly, delayed the increase in ETT cuff pressure during general anesthesia with N_2_O (*P* = 0.029). However, the inhibitory effect in the clinical settings was smaller than that in vitro.

**Conclusions:**

Lubrication of the ETT cuff with K-Y™ jelly may delay the increase in cuff pressure during general anaesthesia with N_2_O. However, the clinical significance of this effect may be limited.

**Trial registration:**

UMIN Clinical Trials Registry: UMIN000031377 on March 1, 2019.

## Implication statement

Lubrication of the ETT cuff with K-Y™ jelly may delay the increase in cuff pressure during general anesthesia with N_2_O. Furthermore, lubricating ETT cuffs with K-Y™ jelly does not affect the incidence of post-operative sore throat and hoarseness.

## Background

Increases in cuff pressure of an endotracheal tube (ETT) due to diffusion of nitrous oxide (N_2_O) into the cuff have been well documented [1, 2]. Cuff pressure should be maintained lower than the capillary perfusion pressure of the tracheal mucosa [3]. Inadvertently high cuff pressure during general anesthesia using N_2_O can cause tracheal morbidity [4, 5]. Furthermore, ETT cuff pressure monitoring during general anesthesia using N_2_O is recommended to ensure that the cuff pressure remains within safe limits to avoid airway morbidity in children [6, 7] and adults [8, 9]. Consequently, monitoring and adjusting the cuff pressure of an ETT during general anesthesia should be performed carefully as standard clinical practice, particularly if N_2_O is used [10].

In our previous study, we found that the application of K-Y™ jelly (Johnson & Johnson, New Brunswick, NJ, USA) on the ETT cuff surface inhibited an increase in cuff pressure during N_2_O exposure in vitro [10]. However, we found several critical differences between our previous laboratory study and existing clinical conditions, which may limit the extrapolation of these results to the clinical setting [10]. We have chosen two major factors whose influences on the effects of lubrication we will assess. First, the amount of K-Y™ jelly retained on the cuff may be reduced during tracheal intubation, and part of the lubricant may be washed out with time during ventilation, suggesting that the inhibitory effect of lubrication may decrease in clinical settings. Second, since all experiments in our previous laboratory study were conducted at a room temperature of 24 °C [10], it is unclear how lubrication influences the N_2_O-induced cuff pressure increase at body temperature. Therefore, in the current study, we sought to study how a reduction in the amount of K-Y™ jelly and a rise in temperature affect the inhibitory effects of lubrication on cuff pressure increase during N_2_O exposure in vitro and to determine whether the inhibitory effects of K-Y™ jelly [10] hold true in clinical settings.

## Methods

### Laboratory study 1

We used a Parker Flex-Tip™ tracheal tube with internal diameter (ID) of 7.5 mm (Parker Medical, Highlands Ranch, CO, USA) and an acrylic cylinder with an ID of 20 mm as a trachea model. To test whether lubrication with K-Y™ jelly inhibits cuff pressure increase during N_2_O exposure in a dose-dependent manner, groups were formed based on the amount of K-Y™ jelly used: 0 g, 1 g, and 3 g for laboratory study 1. Lubrication was performed as described earlier [10]. After adjusting the cuff pressure to 15 mmHg (approximately 20 cmH_2_O), we measured the cuff pressures in the trachea model under the three different lubrication conditions consecutively at a room temperature of 24 °C under continuous flushing with 3 L/min of 66% N_2_O in oxygen at 20 min, 40 min, and 60 min of N_2_O exposure, respectively. After completing the three consecutive measurements of cuff pressure, we calculated the inhibitory rate of 1.0 g and 3.0 g of K-Y™ jelly (%) on the cuff pressure increase as follows: Inhibitory rate of 1.0 g or 3.0 g of K-Y™ jelly (%) = {1 – (cuff pressure with 1.0 g or 3.0 g of K-Y™ jelly at 60 min of N_2_O exposure – 15) mmHg / (cuff pressure without K-Y™ jelly at 60 min of N_2_O exposure – 15) mmHg} × 100 (%). We conducted four sets of the three consecutive measurements. That is, a total number of 12 measurements were performed.

### Laboratory study 2

In a setting similar to laboratory study 1, we further measured the cuff pressures without lubrication and with lubrication with 3.0 g of K-Y™ jelly at 20 min, 40 min, and 60 min of N_2_O exposure, respectively, under the two different temperature conditions (24 and 37 °C) to investigate the effect of temperature on the inhibitory effect of K-Y™ jelly on cuff pressure increase. After adjusting the cuff pressure at 15 mmHg, we measured the cuff pressures with and without lubrication consecutively under the same temperature condition (24 or 37 °C) and under the same gas protocol as described in Laboratory study 1. After completing the two consecutive measurements, we calculated the inhibitory rate (%) of K-Y™ jelly on the cuff pressure increase under the same temperature condition as follows: Inhibitory rate (%) = {1- (cuff pressure with lubrication at 60 min of N_2_O exposure – 15) mmHg / (cuff pressure without lubrication at 60 min of N_2_O exposure – 15)} × 100 (%). We performed six sets of the two consecutive measurements (with and without lubrication) under the same temperature condition (24 or 37 °C). That is, a total of 24 measurements were performed. From April 2018 to August 2018, laboratory studies 1 and 2 were conducted at the Department of Anesthesiology, Mizonokuchi Hospital, Teikyo University School of Medicine, Kawasaki, Japan.

### Clinical study

Our clinical study and protocol were approved by the Tomei Atsugi Hospital Research Committee (Atsugi, Japan; approved on January 11, 2018; Study protocol number: 171205). This study was also registered in the UMIN Clinical Trials Registry (UMIN000031377). From April 2018 to July 2018, after obtaining written informed consent, 50 patients who were scheduled to undergo general anesthesia with tracheal intubation were randomly assigned to one of the two groups: intubation with the ETT lubricated with K-Y™ jelly (25 patients; K-Y™ jelly (+) group) and intubation with the non-lubricated ETT (25 patients; K-Y™ jelly (−) group) at Tomei Atsugi Hospital. Randomization was performed by using computer-generated random numbers. For lubrication, 3.0 g of K-Y™ jelly was applied to the ETT cuff in the K-Y™ jelly (+) group. Inclusion criteria used included patients aged 20–80 years, with ASA physical status I or II, BMI < 35 kg/m^2^, and height of 150 to 180 cm. The exclusion criteria consisted of anticipated difficult airway, a documented history of difficult intubation, cervical spine immobilization, tumors and polyps in the upper airway, potentially high risk of post-operative nausea and vomiting (PONV), and patient refusal. Patients scheduled for major cardiovascular and thoracic surgery, and laparoscopic surgery were also excluded.

A Parker Flex-Tip™ tracheal tube with an ID of 7.5 mm was used for all the patients. After tracheal intubation, the patients’ lungs were ventilated under pressure-controlled ventilation (PCV) with a peak airway pressure of 10–15 cmH_2_O, no positive end-expiratory pressure, and at a frequency of 8–10/min using 6 L/min of 66% N_2_O in oxygen with sevoflurane at end-tidal concentration of 1.5–2.0%. After mechanical ventilation was started, cuff pressures were measured with a pressure transducer (CODAN Xtrans™, CODAN, Lensahn, Germany) connected to the pilot balloon and monitored on an anesthesia monitoring system (BSM-6701, Nihon Kohden, Tokyo, Japan). Immediately after the cuff pressure was adjusted at 15 mmHg, an independent anesthesiologist recorded the time span until the cuff pressure increased to 25 mmHg (clinically safe limit; approximately 33 cmH_2_O). At this point, the cuff pressure was reduced below the clinically safe limit. Anesthesia was maintained using 6 L/min of 66% N_2_O in oxygen with 1.5–2.0% sevoflurane, and with intravenous fentanyl and rocuronium.

At the end of surgery, the administration of sevoflurane and N_2_O were stopped, and the total flow of 100% oxygen was set to 6 L/min. A 200 mg dose of sugammadex was administered, and tracheal extubation was gently performed. All the patients were asked about the presence of sore throat and hoarseness using an established 4-point scale [11, 12] by an independent anesthesiologist about 30 min after extubation at the point of leaving the operating room. Sore throat was graded as: none, mild (less severe than with a cold), moderate (similar to a cold), and severe (more severe than with a cold). Hoarseness was graded as: none, mild (noted only by the patient), moderate (obvious to an observer), and severe (i.e. aphonia).

### Statistical analysis

In the laboratory study 1, a two-way repeated measures ANOVA (time × amount of K-Y™ jelly) followed by Tukey’s test with Bonferroni correction was used to compare the cuff pressure behaviors between the three different lubrication conditions. *P*-values < 0.0167 were considered statistically significant in the post hoc comparison at each time point. A comparison of the inhibitory rates of 1.0 g of K-Y™ jelly (%) with those of 3.0 g of K-Y™ jelly (%) was then performed using the Mann-Whitney U-test. In laboratory study 2, a two-way repeated ANOVA (time × lubricant) followed by an unpaired t-test with Bonferroni correction was used to compare the cuff pressure behaviors with and without lubrication under the same temperature condition. *P*-values < 0.0167 were considered statistically significant in the post hoc comparison at each time point. A comparison of the inhibitory rates of K-Y™ jelly (%) between the two different temperature conditions was then performed using the Mann-Whitney U-test.

In the clinical study, we used an unpaired t-test for the comparison of the duration until the upper limit of cuff pressure (25 mmHg) was reached. The incidence of sore throat and hoarseness were analyzed using Chi-squared tests. Patients’ characteristics were compared using unpaired t-tests and Chi-squared tests where appropriate. The sample size was calculated based on the measurements from our pilot study of the duration until the cuff pressure of 25 mmHg was reached. On the basis of an expected maximum SD of 13.7 min, 21 patients in each group were required to demonstrate a 30% difference in that time span between groups (β = 0.2; α = 0.05).

## Results

**Fig. 1:**
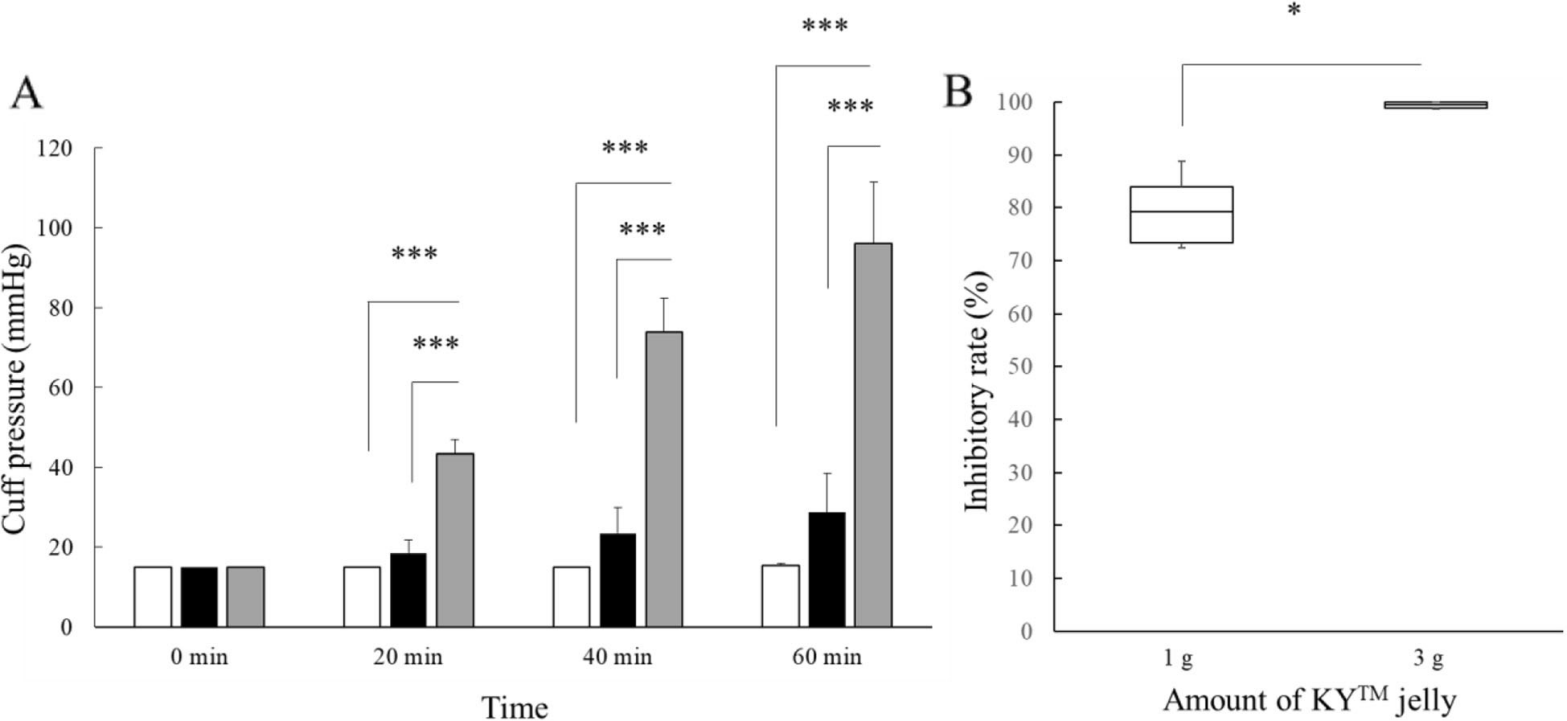
Graph **a** shows the comparison of cuff pressures between the three different lubrication conditions (0 g, 1.0 g and 3.0 g of K-Y™ jelly) under continuous flushing with nitrous oxide. Grey, black, and white bars in Graph **a** represent the cuff pressures with lubrication with 0 g, 1.0 g and 3.0 g of K-Y™ jelly. Data are expressed as mean (SD) in Graph **a**. *P*-values < 0.0167 (0.05/3) were considered statistically significant in the post hoc comparison of the three groups at each time point. Graph **b** shows the comparison of the inhibitory rates (%) of 1.0 g K-Y™ jelly with those of 3.0 g K-Y™ jelly. The horizontal bars, the boxes, and the whiskers represent the median, IQR, and ranges, respectively in Graph B. *P*-value < 0.05 was considered statistically significant. *P < 0.05 and ***P < 0.0001

In laboratory study 1, the interaction between time and the amount of K-Y™ jelly was significant (*P* < 0.0001, F = 36.6). The cuff pressures with 1.0 g and 3.0 g of K-Y™ jelly were significantly lower than those without K-Y™ jelly at 20 min, 40 min, and 60 min of N_2_O exposure (*P* < 0.0001, *P* < 0.0001 and *P* < 0.0001, respectively; Fig. 1a). Although no significant differences in cuff pressure were found between 1.0 g of K-Y™ jelly and 3.0 g of K-Y™ jelly at any time point (Fig. 1a), the inhibitory rates of 3.0 g of K-Y™ jelly (Median: 99.5%) were significantly greater than those of 1.0 g of K-Y™ jelly (Median: 79.25%) (*P* = 0.02, Fig. 1b).

**Fig. 2:**
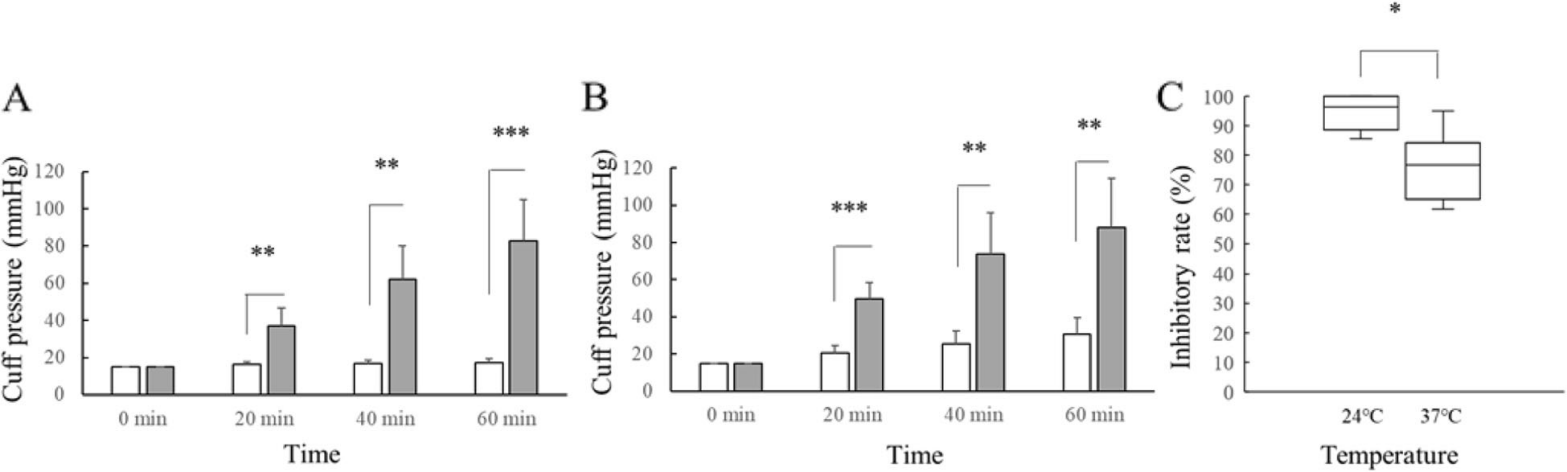
Graphs **a** and **b** show the cuff pressures with (white bar) and without K-Y™ jelly (grey bar) at 24 °C and 37 °C, respectively. Data are expressed as mean (SD) in Graphs **a** and **b**. *P*-values < 0.0167 (0.05/3) were considered statistically significant in the post hoc comparison between the two groups at each time point. Graph **c** shows the comparisons of inhibitory rates (%) of K-Y™ jelly at 24 °C with those at 37 °C. The horizontal bars, the boxes, and the whiskers represent the median, IQR, and ranges, respectively in Graph C. *P*-value < 0.05 was considered statistically significant. *P < 0.05, **P < 0.001 and ***P < 0.0001

In laboratory study 2, the interaction between time and lubricant usage was significant at 24 °C (*P* < 0.0001, F = 21.2) and 37 °C (*P* < 0.0001, F = 11.3). Cuff pressures without lubricant were significantly higher than those with lubricant at each time point of N_2_O exposure at 24 °C (*P* < 0.001, *P* < 0.001 and *P* < 0.0001) and at 37 °C (P < 0.0001, *P* < 0.001 and *P* < 0.001) (Fig. 2a and b). Furthermore, the inhibitory rates of K-Y™ jelly (%) at 24 °C were significantly greater than those at 37 °C (Median: 96.35% vs 76.65%, *P* = 0.019, Fig. 2c).

**Fig. 3:**
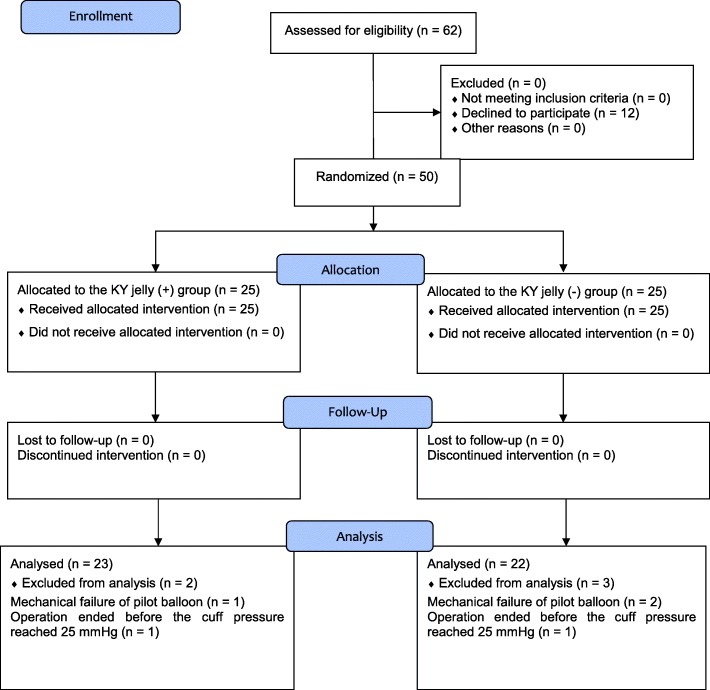
CONSORT flow chart for patients’ recruitment

Patient recruitment and flow in the clinical study are shown in Fig. 3. Fifty patients were enrolled in this study. After exclusion due to some reasons, data were analyzed from 23 patients in the K-Y™ jelly (+) group and 22 patients in the K-Y™ jelly (−) group (Fig. 3). Patient characteristics were similar, and there were no significant differences between groups (Table 1). The time span until the cuff pressure increased to 25 mmHg was significantly longer in the K-Y™ jelly (+) group compared to the K-Y™ jelly (−) group (Table 2, *P* = 0.029). However, no significant differences were found in the incidence of sore throat and hoarseness between groups (Table 3).

**Table 1:** Patient characteristics

	KY jelly (+) *n* = 23	KY jelly (−) *n* = 22
ASA classification (I/II)	13/10	13/9
Sex (male/female)	16/7	18/4
Age (years)	59.5 (13.8)	57.5 (15.5)
Height (cm)	164.0 (8.6)	166.3 (8.4)
Weight (kg)	62.3 (10.5)	66.0 (10.8)
Duration of anesthesia (min)	137.9 (69.2)	125.6 (40.9)

Data are expressed as mean (SD), or numbers of patients. There were no significant differences between groups

**Table 2:** Comparison of the time to reach a cuff pressure of 25 mmHg between the two groups

	K-Y™ jelly (+) group *n* = 23	K-Y™ jelly (−) group *n* = 22	*P*- value
Time (min)	42.5 (15.8)	34.1 (13.1)	0.029*

Data are expressed as mean (SD). *P-value < 0.05 was statistically significant

**Table 3:** Post-operative sore throat and hoarseness in K-Y™ jelly (+) and K-Y™ jelly (−) groups

	K-Y™ jelly (+) group *n* = 23	K-Y™ jelly (−) group *n* = 22	*P*- value
Sore throat (none/mild/moderate/severe)	19/4/0/0	14/8/0/0	0.15
Hoarseness (none/mild/moderate/severe)	12/6/5/0	15/5/2/0	0.43

Values are numbers. There were no significant differences between groups

## Discussion

The increase in ETT cuff pressure due to N_2_O diffusion is a well-known risk during general anesthesia using N_2_O [13–15]. N_2_O diffusion induces hyperinflation of ETT cuff, causing an increased risk of tracheal barotrauma [4, 5]. Therefore, ETT cuff pressure increase when using N_2_O for general anesthesia has been one of the major concerns in clinical practice. Previous reports described some methods to avoid the increase in ETT cuff pressure during N_2_O anesthesia. Combes X et al. [4] reported that filling ETT cuff with saline prevented the cuff pressure increase during N_2_O anesthesia. Furthermore, Karasawa F et al. [16] demonstrated that inflating ETT cuff with a N_2_O gas mixture prevented such increase. However, these methods are not common in clinical practice.

In the current and previous study [10], we used K-Y™ jelly instead of other lubricants that are also used in clinical practice, because K-Y™ jelly appears to be the most frequently used lubricant in a clinical setting in Japan. Our previous study [10] demonstrated that lubrication with K-Y™ jelly strongly and significantly inhibited the N_2_O-induced cuff pressure increase in the laboratory study at a room temperature of 24 °C. Additionally, we raised the possibility that the layer of glycerine-based K-Y™ jelly reduced the diffusion of N_2_O into the ETT cuff [10]. In the current laboratory studies, we found that the inhibitory effect on the ETT cuff pressure increase was reduced when the amount of K-Y™ jelly was reduced and when temperature was raised to body temperature, suggesting such inhibitory effects may decrease in clinical settings. In the current clinical study, we found that lubrication with K-Y™ jelly significantly prolonged the time span to reach the upper safety limit of the cuff pressure by approximately 8 min during general anesthesia using N_2_O in adult patients. To the best of our knowledge, this is the first clinical trial investigating how lubrication with K-Y™ jelly affects the ETT cuff pressure behavior in patients during general anesthesia using N_2_O.

Regarding the protocol of the clinical study, we used an ID 7.5 ETT for adult patients with height 150 to 180 cm regardless of sex. When an ETT cuff is exposed to N_2_O, the rate of diffusion of N_2_O into the ETT cuff is proportional to the surface area available for N_2_O diffusion [17]. Therefore, we used ETTs of the same size for all the patients to standardize the surface area of the ETT cuff. We defined < 25 mmHg (approximately 33 cmH_2_O) as a clinically safe limit, because Seegobin et al. [3] demonstrated that a reduction in tracheal mucosal blood flow in humans begins with ETT cuff pressures greater than 30 cmH_2_O.

In the current study, we found that the cuff pressure of ETTs lubricated with K-Y™ jelly did not increase as rapidly as did those without lubrication used in adult patients during general anesthesia using N_2_O. However, the magnitude of the inhibitory effect on the ETT cuff pressure increase appeared much milder in clinical situations than in vitro [10]. This may be due to several reasons. First, the amount of K-Y™ jelly may be reduced when the ETT cuff is advanced into the oral cavity and trachea during tracheal intubation. Second, due to an increase in the gas permeability of N_2_O in the K-Y™ jelly with temperature, such inhibitory effects may be smaller at body temperature, compared with those at room temperature [10]. In addition to these two factors examined in the current study, a number of factors may constitute possible reasons for the difference between the clinical and laboratory studies. For example, N_2_O diffusion occurs through the tracheal wall membrane in clinical settings [8]. Therefore, the effect of K-Y™ jelly on inhibiting N_2_O diffusion into the ETT cuff may be smaller in clinical settings than in vitro. This is because the layer of K-Y™ jelly on the surface of the ETT cuff facing the mucosal membrane of the trachea may be thinner than that on the carina side of the ETT cuff after tracheal intubation. Furthermore, the tracheal wall in humans is much more elastic than the trachea model used in our previous study [10] and in the current laboratory studies. Therefore, the cuff pressures in the patients’ trachea might not increase as rapidly as did those in vitro. Additionally, we could not exclude the possibility that K-Y™ jelly may be washed out by mucus derived from the tracheal membrane and by the moisture contained in the anesthesia circuit, as it is a water-soluble lubricant. However, it has been shown that the inhibition of fluid leakage across tracheal tube cuffs by K-Y™ jelly lasts for about 24 h in adult patients [18]. This finding suggests that part of K-Y™ jelly may remain on the cuff for much longer than the duration required for common surgical procedures in the clinical settings.

In the current clinical study, we found a significant difference of approximately 8 min in the time span until the cuff pressure increased to 25 mmHg (clinically safe limit) between the two groups. By lubricating the ETT cuff with K-Y™ jelly, increasing the time until the cuff pressure reaches the clinically safe limit by 8 min may have limited its clinical significance because this does not eliminate the necessity of cuff pressure management during general anesthesia using N_2_O. However, the results suggest that lubrication of the ETT cuff may decrease the frequency of needing to adjust the cuff pressure during surgery. Theoretically, the average number of events to adjust the cuff pressure required during a 3-h anesthesia period will be 4.2 and 5.3 times with and without the lubricant, respectively. Consequently, this effect may lead to a reduction in the incidence of complications associated with cuff pressure manipulation, such as cough reflex, bucking, and hemodynamic responses. Therefore, the rate of increase in cuff pressure has clinical meanings in general anesthesia using N_2_O.

Regarding the incidence of sore throat and hoarseness, there was no significant difference between the two groups in the current study. Sumathi et al. [19] reported that the incidence of sore throat was significantly less when lidocaine jelly was applied on the cuff, compared with that when no jelly was applied. However, it has also been reported that the application of lidocaine jelly increases post-operative sore throat [20, 21]. Furthermore, Doukumo et al. [22] reported that K-Y™ jelly is superior to lidocaine jelly in preventing post-operative sore throat and hoarseness. Consequently, the effect of lubrication of the cuff on the incidence of sore throat and hoarseness still remains controversial. In the current study, we found that lubricating the ETT cuff with K-Y™ jelly had no effect on the incidence of post-operative sore throat and hoarseness.

The current study has some limitations. First, we measured the actual values of the ETT cuff pressure during 60 min of N_2_O exposure in current laboratory studies and our previous study [10]. Conversely, we measured the time span until the cuff pressure increased to 25 mmHg in the clinical study. That is, the measured parameters were different between laboratory and clinical studies, suggesting that the direct comparison of the results between laboratory and clinical studies may not be feasible However, for ethical reasons and patient safety, we could never leave the ETT cuff pressure increase above the clinically safe limit. Second, we did not test whether other lubricants also have an inhibitory effect on ETT cuff pressure increase during N_2_O exposure. Thus, it is unclear whether other lubricants have a similar effect. Third, we assessed post-operative sore throat and hoarseness only at the point of leaving the operating room. Previous studies evaluated sore throat and hoarseness at the various time points after general anaesthesia [19–23]. Evaluation at various time points after complete recovery of consciousness may be more informative. Furthermore, the scales used in the evaluation of sore throat and hoarseness were subjective. However, an independent anesthesiologist asked all the patients about sore throat and hoarseness, and scored based on the agreed scale, suggesting that our scoring system was standardized, and the results are reliable.

## Conclusions

Lubrication of ETT cuffs with K-Y™ jelly may delay the increase in cuff pressure during general anaesthesia using N_2_O. However, the inhibition of the N_2_O-induced cuff pressure increase appeared smaller in clinical settings than in laboratory studies and is of limited clinical significance. Furthermore, lubricating ETT cuffs with K-Y™ jelly does not affect the incidence of post-operative sore throat and hoarseness.

## Data Availability

The datasets analyzed during the current study are available from the corresponding author on reasonable request.

## Abbreviations

ETT: Endotracheal tube
ID: Internal diameter
N_2_O: Nitrous oxide

## References

[1] Stanley TH, Kawamura R, Graves C (1974). Effects of nitrous oxide on volume and pressure of endotracheal tube cuffs. Anesthesiology.

[2] Karasawa F, Ohshima T, Takamatsu I, Ehata T, Fukuda I, Uchihashi Y, Satoh T (2000). The effect on intracuff pressure of various nitrous oxide concentrations used for inflating an endotracheal tube cuff. Anesth Analg.

[3] Seegobin RD, van Hasselt GL (1984). Endotracheal cuff pressure and tracheal mucosal blood flow: endoscopic study of effects of four large volume cuffs. Br Med J (Clin Res Ed).

[4] Combes X, Schauvliege F, Peyrouset O (2001). Intracuff pressure and tracheal morbidity: influence of filling with saline during nitrous oxide anesthesia. Anesthesiology.

[5] Tu HN, Saidi N, Leiutaud T, Bensaid S, Menival V, Duvaldestin P (1999). Nitrous oxide increases endotracheal cuff pressure and the incidence of tracheal lesions in anesthetized patients. Anesth Analg.

[6] Bernet V, Dullenkopf A, Cannizzaro V, Stutz K, Weiss M (2006). An in vitro study of the compliance of paediatric tracheal tube cuffs and tracheal wall pressure. Anaesthesia..

[7] Dullenkopf A, Gerber A, Weiss M (2004). The microcuff tube allows a longer time interval until unsafe cuff pressures are reached in children. Can J Anaesth.

[8] Dullenkopf A, Gerber AC, Weiss M (2004). Nitrous oxide diffusion into tracheal tube cuffs: comparison of five different tracheal tube cuffs. Acta Anaesthesiol Scand.

[9] Tsuboi S, Miyashita T, Yamaguchi Y, Yamamoto Y, Sakamaki K, Goto T (2013). The TaperGuard™ endotracheal tube intracuff pressure increase is less than that of the hi-lo™ tube during nitrous oxide exposure: a model trachea study. Anesth Analg.

[10] Koyama Y, Oshika H, Nishioka H, Kamoshida N, Tanaka S, Inagawa G, Andoh T (2018). K-Y jelly inhibits increase in endotracheal tube cuff pressure during nitrous oxide exposure in vitro. BMC Anesthesiol.

[11] Stout DM, Bishop MS, Dwersteg JF, Cullen BF (1987). Correlation of endotracheal tube size with sore throat and hoarseness following general anesthesia. Anesthesiology.

[12] Koyama Y, Nishihama M, Inagawa G, Kamiya Y, Miki T, Kurihara R, Goto T (2011). Comparison of haemodynamic responses to tracheal intubation using the airway scope(®) and Macintosh laryngoscope in normotensive and hypertensive patients. Anaesthesia.

[13] Stanley TH (1974). Effects of anesthetic gases on endotracheal tube cuff gas volumes. Anesth Analg.

[14] Bernhard WN, Yost LC, Turndorf H, Cottrell JE, Paegle RD (1978). Physical characteristics of and rates of nitrous oxide diffusion into tracheal tube cuffs. Anesthesiology..

[15] Stanley TH (1975). Nitrous oxide and pressures and volumes of high- and low-pressure endotracheal-tube cuffs in intubated patients. Anesthesiology..

[16] Karasawa F, Tokunaga M, Aramaki Y, Shizukuishi M, Satoh T (2001). An assessment of a method of inflating cuffs with a nitrous oxide gas mixture to prevent an increase in intracuff pressure in five different tracheal tube designs apparatus. Anaesthesia..

[17] Kim JM, Mangold JV, Hacker DC (1985). Laboratory evaluation of low-pressure tracheal tube cuffs: large-volume v. low-volume. Br J Anaesth.

[18] Blunt MC, Young PJ, Patil A, Haddock A (2001). Gel lubrication of the tracheal tube cuff reduces pulmonary aspiration. Anesthesiology.

[19] Sumathi PA, Shenoy T, Ambareesha M, Krishna HM (2008). Controlled comparison between betamethasone gel and lidocaine jelly applied over tracheal tube to reduce postoperative sore throat, cough, and hoarseness of voice. Br J Anaesth.

[20] Klemola UM, Saarnivaara L, Yrjölä H (1988). Post-operative sore throat: effect of lignocaine jelly and spray with endotracheal intubation. Eur J Anaesthesiol.

[21] Lee J, Lee YC, Son JD, Lee JY, Kim HC (2017). The effect of lidocaine jelly on a taper-shaped cuff of an endotracheal tube on the postoperative sore throat: a prospective randomized study: a CONSORT compliant article. Medicine (Baltimore).

[22] Doukumo D, Faponle A, Adenekan A, Olateju S, Bolaji B (2011). Effects of lidocaine and k-y jellies on sore throat, cough, and hoarseness following endotracheal anaesthesia. J West Afr Coll Surg.

[23] Soltani HA, Aghadavoudi O (2002). The effect of different lidocaine application methods on postoperative cough and sore throat. J Clin Anesth.

